# Holder design for robotic assisted ultrasound and MRI imaging guided needle biopsy

**DOI:** 10.1186/2050-5736-3-S1-P82

**Published:** 2015-06-30

**Authors:** Ting Zhang

**Affiliations:** 1 University of Dundee, Dundee, United Kingdom

## Background/introduction

Ultrasound and MRI imaging guiding system for Robotic assisted interventional procedures such as needle biopsy and FUS ablation have to be improved to allow a one stop shop multimodality image guidance. A specific holder which has the capability for connecting the application module of the interventional robotic system “INNOMOTION” (IBSmm, CZ) with SIEMENS wireless ultrasound probe (Acuson Freestyle) was designed and manufactured in order to achieve the desired function. The work is a subproject in FUTURA an EU FP7 funded project for the development of robotic assisted Ultrasound guided focused ultrasound.

## Methods

The software SOLIDWORKS is mainly employed for the design and simulation. At the first step, details of the dimensions of the application module of the robot arm should be obtained. Moreover, the motion range should be considered as well as was tested in a mock up situation. Ultrasound positioning, organ tracking and focused ultrasound application have been developed on *ex vivo* animal organ models and Thiel soft embalmed human cadavers. Cadaver work has been approved by the local IRB TAC according to rules of the Scottish anatomy act. The holder was designed to meet the requirements listed below:

1. As the application module of coaxial probes provides two degrees of freedom and is attached to a robotic arm with five degrees of freedom, which assures stable positioning of the instrument within a tool center point that keeps the ‘‘invariant point of insertion’’ at the skin entry point, the design should not change the default attached position of the needle.

2. The position of the probe should be adjustable according to the actual operating environment, which mainly includes the horizontal, vertical and axial translation and angle rotation.

3. As the skin level will supply a pushing force to the probe, certain spring should be operated to provide the reacting force in order to keep the working position of the probe.

4. The holder should allow easy exchange of probes for both diagnostic and focused ultrasound

Furthermore, as the holder should be employed in the MRI working area, ABS plastic is picked for the fabrication material and 3D printing technology has been employed.

## Results and conclusions

Results: The holder can be separated into seven parts: hangers, main frame, horizontal slide, connector of horizontal and rotational block, rotational block, axial slide and the probe connector. Moreover, the dimension is approximately 200mm long, 75mm wide and 140mm high, and the sketch diagram is in the imgage attached. The practical effect drawings are displayed in the image attached as well. The robotic arm is roughly simulated by the cylinder bar as that part is not so relative to the holder while the application module at the top of the arm is precise. Basically, when it comes to the actual operation, the motion of the application module is combined by movements forward and backward and rotation right and left. The full function can be achieved when the manual translation and rotation of the cannulae is added.

Conclusion: The final holder is currently being manufactured in the 3D printer and has met all the requirements needed for the desired function considering the limited assembly space. For instance, the needle or probe is located in the default spot for the reason that the skin entry point can be confirmed by the existing program; the relative location between the probe and the needle as well as the angle of the probe can be adjusted for the best imaging view; the axial location of the probe can be regulative for better effect with the specific spring. Therefore this design does have the capability to improve robotic positioning of ultrasound during MRI imaging guided robotic assisted needle biopsy procedures and provides a fundamental concept for the advanced precise instruments in this area.

**Figure 1:**
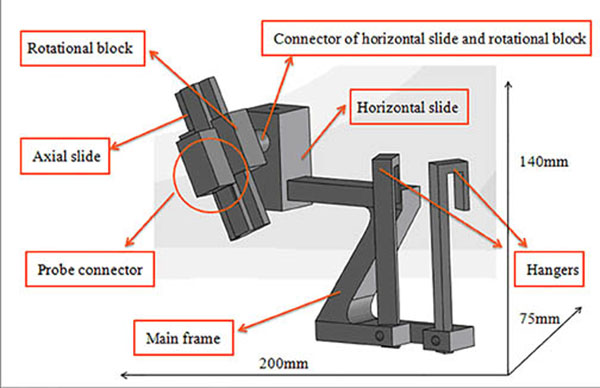
Sketch diagram

**Figure 2:**
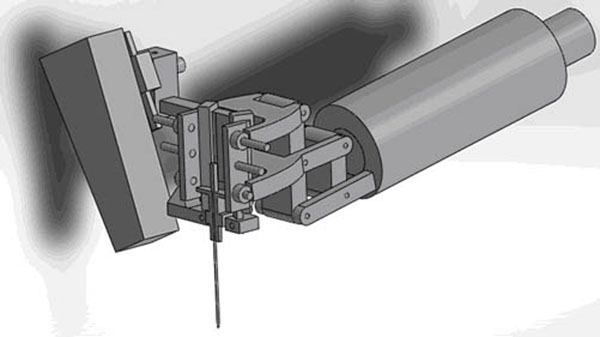
Effect drawing 1

**Figure 3:**
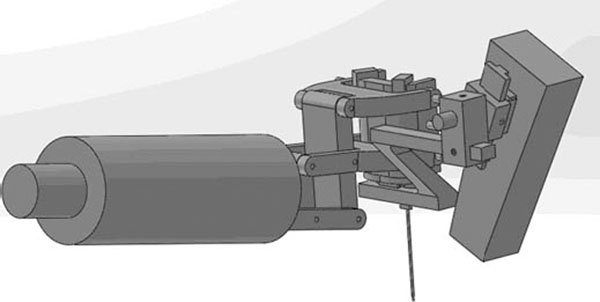
Effect drawing 2

**Figure 4:**
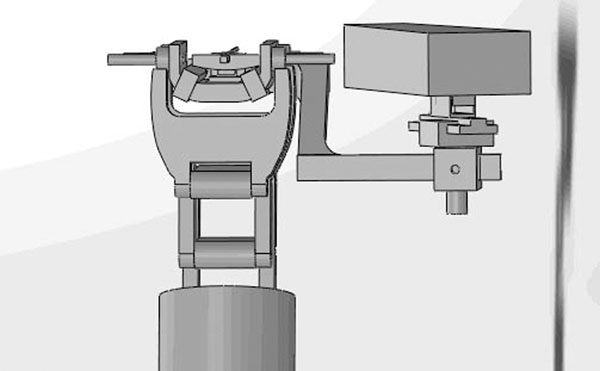
Effect drawing 3

